# Supplementary material for: Activation of Bradykinin B2 Receptors in Astrocytes Stimulates the Release of Leukemia Inhibitory Factor for Autocrine and Paracrine Signaling
Source: Int J Mol Sci. 2024 Dec 5;25(23):13079. doi: 10.3390/ijms252313079 (PMC11642044; doi:10.3390/ijms252313079)
Supplement: Supplementary file 1 [file ijms-25-13079-s001.zip › ijms-3262157-supplementary.pdf]

Supplementary Methods (for Supplementary Figures)

Transfection and conditioned media preparation: For the transfection issue, all Gα subunits cDNAs (i.e. $G\alpha_q$, $G\alpha_{11}$, $G\alpha_{16}$, $G\alpha_S$, $G\alpha_{i3}$, $G\alpha_{oA}$, $G\alpha_Z$, and $G\alpha_{12}$) obtained from cDNA Resource Center (Bloomsburg, PA) were transfected by using Lipofectamine™ 2000 transfection reagents according to the supplier's instruction (Thermo Fisher Scientific, Waltham, MA); 1 µg of each Gα subunit cDNA (wildtype or activated mutant) was introduced individually to each well (70-80% confluence of HEK293 cells) in 6-well plates. Transfected cells were maintained in normal growth medium (2 mL of MEM per well with 10% FBS) for 24 h, followed by serum starvation (1 mL of MEM per well without FBS) for 24 h. The resulting conditioned media were collected in 1.5 mL microcentrifuge tubes, with the presence of secretory cytokine (e.g. LIF, IL-6, CT-1, OSM, and CNTF) quantified by cytokine multiplex assay kits (Merck Millipore, MA, USA). HEK293 cells (80-100% confluence in 6-well plates) stably expressing MT_1R alone or together with $G\alpha_{16}$ as described previously [Ref. 21] were treated with 1 µM of melatonin (Tocris Bioscience, Minneapolis, MN) in 1 mL of MEM per well without FBS for 24 h. The resulting conditioned media were collected in 1.5 mL microcentrifuge tubes, with the presence of secretory LIF and IL-6 quantified by ELISA assays (Abcam, Cambridge, UK) according to the manufacturer's instructions.

Cytokine multiplex assay kits and ELISA: The quantitative measurement of IL-6 family cytokines (e.g. IL-6, LIF, CNTF, CT-1, OSM) was performed by using the Merck Millipore Multiplex Assay as previously described [21,23]. Briefly, a bead mixture with various capture antibodies (50 µL) was added to the 96-well microtitre plate, followed by 50 µL of cytokine standard mix or test sample and then 25 µL of a biotinylated detection antibody mixture. The mixtures were incubated for 30 min at 25°C with gentle shaking. Unbound antibodies were removed by three gentle washes. Finally, 50 µL of streptavidin-PE solution was added and incubated for another 30 min with gentle shaking. The detection beads were then washed again and resuspended in 120 µL of reading buffer. Bio-Plex™ 200 system (Bio-Rad Laboratories) with the Bio-Plex manager software (version 5.0) was utilized for signal detection and data analysis. Curve fitting was applied to each standard curve according to the manufacturer's manual and sample concentrations were interpolated from the standard curves. Individual detections for LIF and IL-6 were performed by using the corresponding ELISA kits from Abcam (Cambridge, UK) according to the manufacturer's instructions.

Immunofluorescence and Western blot: Primary neurons or astrocytes were cultured on 18 mm round microscope cover slips at a density of $2x10^5$ cells/mL for neurons or $1x10^5$ cells/mL for astrocytes. After the cells were adhered to the coverslips, the culture medium was aspirated, and coverslips were rinsed with Tris-buffered saline (TBS) twice. The cells were then incubated with formaldehyde for 20 min for fixation. After washing twice with TBS, the coverslips were transferred to a parafilm plate and incubated with 0.1% Triton-X in TBS for 5 min to facilitate permeabilization. Subsequently, the coverslips were rinsed with TBS and blocked using 5% BSA in TBS at room temperature for 1 h. Following blocking, the coverslips were exposed to primary antibodies overnight at 4° C. On the next day, the coverslips underwent three washes with TBS before being incubated with secondary antibodies at room temperature for 1 h in darkness. For astrocytic culture, primary antibodies such as rabbit NeuN antibody (Merck Millipore, Cat. No. MABN140, 1:100 dilution) and mouse GFAP antibody (abcam, Cat. No. ab4648, 1:100 dilution) were accompanied with Alexa Fluor 488 (green) anti-rabbit IgG (Cell Signaling Technology, Cat. No. #4412, 1:500 dilution) and Alexa Fluor 555 (red) anti-mouse IgG (Cell Signaling Technology, Cat. No. #4409, 1:500 dilution), respectively, as the secondary antibodies. While for neuronal culture, primary antibodies such as rabbit GFAP antibody (abcam, Cat. No. ab7260, 1:100 dilution) and mouse MAP2 antibody (abcam, Cat. No. ab11268, 1:100 dilution) were accompanied with Alexa Fluor 488 (green) anti-rabbit IgG and Alexa Fluor 555 (red) anti-mouse IgG, respectively. After washing with TBS three times, coverslips were immersed in TBS containing DAPI for 15 min and then washed again with TBS. Subsequently, the coverslips were inverted onto slides and allowed to air-dry at room temperature overnight in darkness before mounting with Prolong gold (ThermoFisher, Cat. No. P36930). On the third day, microscopic observation was performed with Zeiss LSM 510 META, and images were deconvoluted with LSM Image Browser Rel. 4.2 (Carl Zeiss, Oberkochen, Germany).

For the Western blots, GFAP and β_{III}-tubulin detections for mouse primary neuronal / astrocytic cultures were performed by using the GFAP antibody (abcam, Cat. No. ab7260, 1:1000 dilution) and the β_{III}-tubulin antibody (Cell Signaling Technology, Cat. No. #5568, 1:1000 dilution), respectively, followed by horseradish peroxidase-conjugated secondary antisera. Immunoblots were visualized by chemiluminescence with the WesternBright ECL reagents from Advansta (San Jose, CA, USA). Chemiluminescence signals were then detected by the ChemiDoc Imaging Systems (Bio-Rad, Hercules, CA).

Detection of bradykinin-induced activation of MAPK subtypes: U87-MG cells were stimulated with increasing duration (0, 5, 10, 15, 30, 45 min) of bradykinin (1 μM), with the protein content in the subsequent cell lysates resolved in SDS-PAGE, followed by immunodetection for various MAPK subgroups including ERK, p38, and JNK, with specific antibodies which recognize either their stimulatory phosphorylated forms or their total amount of protein levels. U87-MG cells were also pretreated with or without the B_2R-specific antagonist (HOE-140, 5 μM for 15 min) prior to stimulation with bradykinin (1 μM, 10 min), with the subsequent cell lysates subjected to similar detections of MAPK subgroups.

RT-PCR: For mRNA detection, the total RNA of the cells was extracted using Trizol reagent. One microgram of total RNA was reversely transcribed using cDNA synthesis kits for reverse transcription PCR (RT-PCR) according to the manufacturer's protocol. The cDNA was amplified using the following primers: human LIFR-forward (5'-CGGGAGCGTACCGACTGAC-3') and human LIFR-reverse (5'-AGCCACTGGAAATTTGAAGCAGT-3'), mouse LIFR-forward (5'-TCCAAGGACGGAACCAGTAG-3') and mouse LIFR-reverse (5'- TGCCATCGAATGCTCACCG-3'), GAPDH-forward (5'-AAGTTGTCATGGATGACCTTGGC-3') and GAPDH-reverse (5'-GGCGTCTTCACCACCATGGAG-3'). The PCR amplifications were performed at 95°C for 3 min, followed by 35 cycles of thermal cycling at 98°C for 20 sec, 60°C for 15 sec and 72°C for 15 sec, and a final extension at 72°C for 15 sec. GAPDH was used as an endogenous control to normalize differences. After PCR amplification, 5 μl of the PCR products were used for electrophoresis on 1.5% agarose gels and stained with Midori Green. All primers utilized in our current study were designed by using Primer-BLAST (NIH, USA).

Detection of endogenous LIF receptor (LIFR): Cell lysates (with 25 μg of protein per samples) of human origin (U87-MG cells, SK-N-MC cells, MCF-7 cells) and murine origin (NIH-3T3 cells, primary astrocytes and primary neurons) were subjected to SDS-PAGE with the resolved proteins transferred to a nitrocellulose membrane, followed by immunodetection of endogenously expressed LIFR with a LIFR-specific antibody (abcam, Cat. No. ab101228, 1:1000 dilution).

Statistical analysis: Experimental data were statistically analyzed by GraphPad Prism version 7.0 for PC (GraphPad Software, CA, USA). The data were analyzed using one-way analysis of variance (ANOVA) test, then further compared to basal or control group by using Dunnett's test. P-values of less than 0.05 were defined as statistically significant. Independent experiments were typically performed in triplicates with the number of repeats (n values) indicated in the corresponding figure legends.

Supplementary Fig. S1

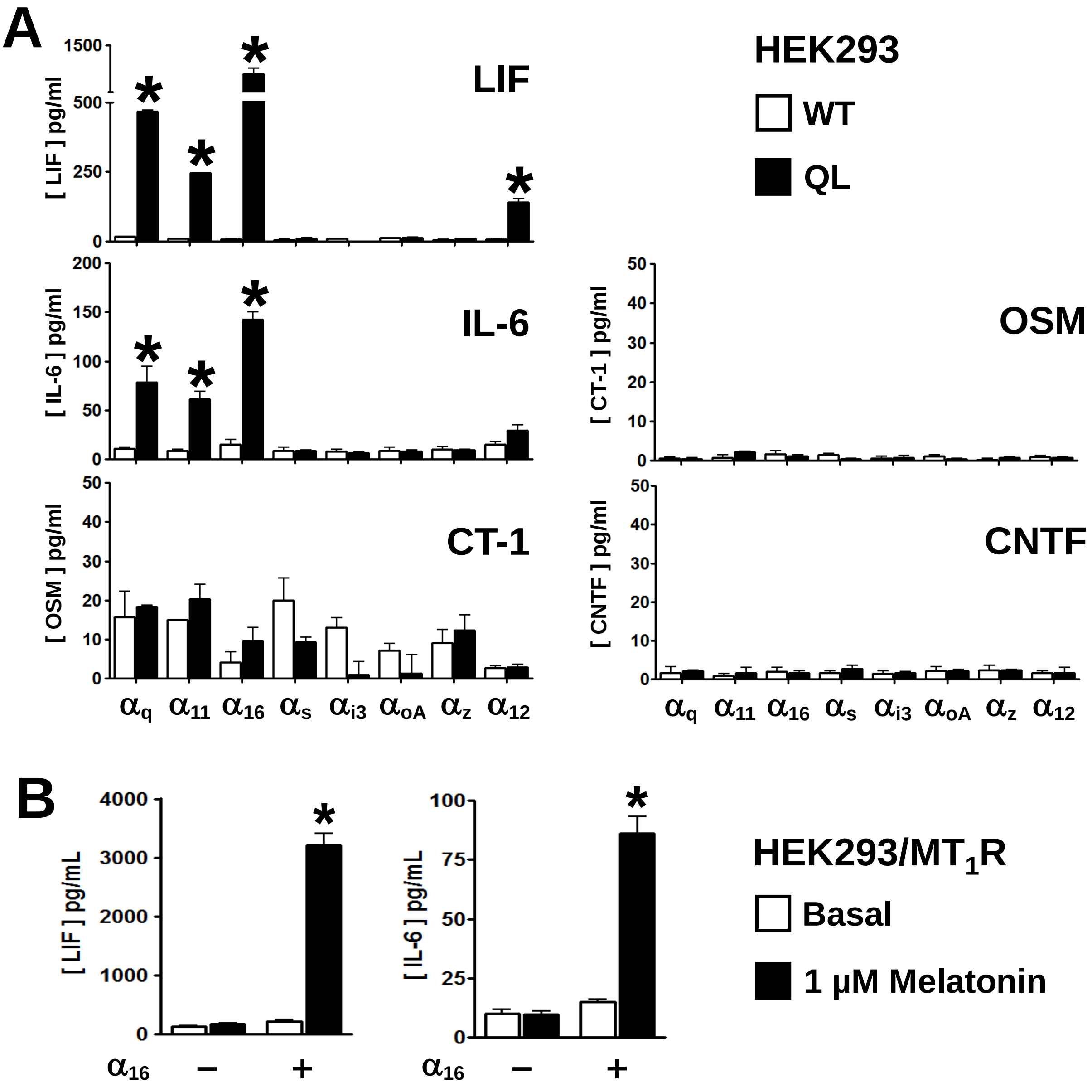

Supplementary Figure S1. G protein / GPCR regulation of cytokine secretion. (A) Constitutive activation of specific Gα subunits resulted in elevated production of LIF and IL-6, but not CT-1, OSM or CNTF. HEK293 cells were transiently transfected with the wild-type (WT) or the constitutively activated mutant (QL) of various Gα subunits. The corresponding conditioned media were collected and subjected to the detection of secretory LIF, IL-6, CT-1, OSM and CNTF by cytokine multiplex assay kits. *Activated mutants of Gα subunits triggered significant production of LIF and IL-6 (* $p < 0.05$; n=3). Open bars and filled bars represent WT and OL, respectively. (B) Functional coupling between MT_1R and G_{16} results in elevated LIF and IL-6 productions. HEK293 cells stably expressing MT_1R alone or together with $G\alpha_{16}$ were stimulated with 1 µM melatonin in the absence of serum for 24 h, the resulting conditioned media were subjected to ELISA for the quantification of LIF and IL-6. *Agonist treatments significantly increased the production of LIF and IL-6 (* $p < 0.05$; n = 3).

Supplementary Fig. S2

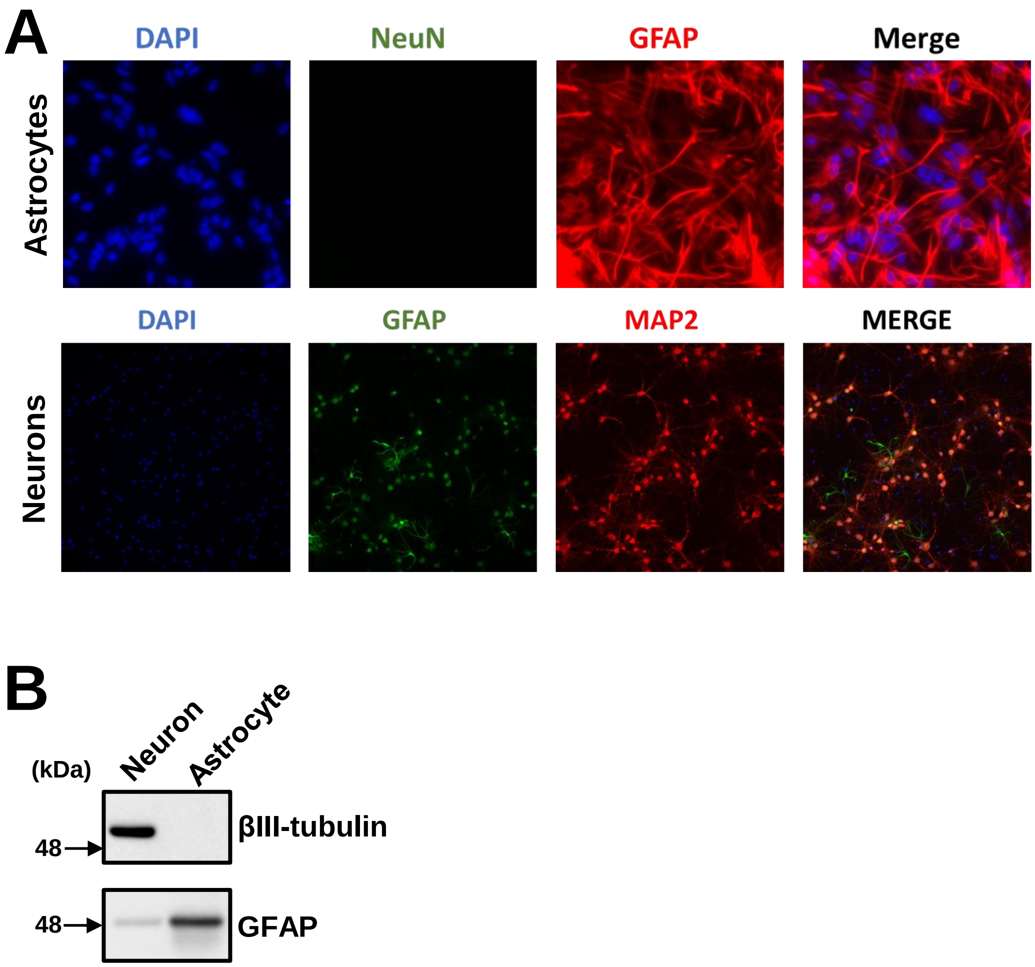

Supplementary Figure S2. Preparation of murine primary astrocytes and neurons. Murine primary astrocyte cultures from E16.5-17.5 wild-type C57BL/6J mice were prepared from cerebral cortexes of neonatal mice (P2), while murine primary cortical neuronal cultures were obtained from cerebral cortices of E16 ICR mouse embryos. (A) Immunostaining was performed with specific antibodies which recognized astrocytic markers (GFAP) and neuronal markers (NeuN and MAP2), respectively. The primary astrocytic culture was of high purity as indicated by a strong GFAP immuno-fluorescent signal without detectable NeuN, while the primary neuronal culture was associated with a readily detectable MAP2 immuno-fluorescent signal and a weak GFAP readout, as a complete absence of astrocytes is generally unfavorable for normal neuronal survival. (B) Lysates of murine primary neurons and astrocytes were subjected to SDS-PAGE, followed by immunodetection of β_{III}-tubulin (neuronal marker) and GFAP (astrocytic marker) with specific antibodies.

Supplementary Fig. S3

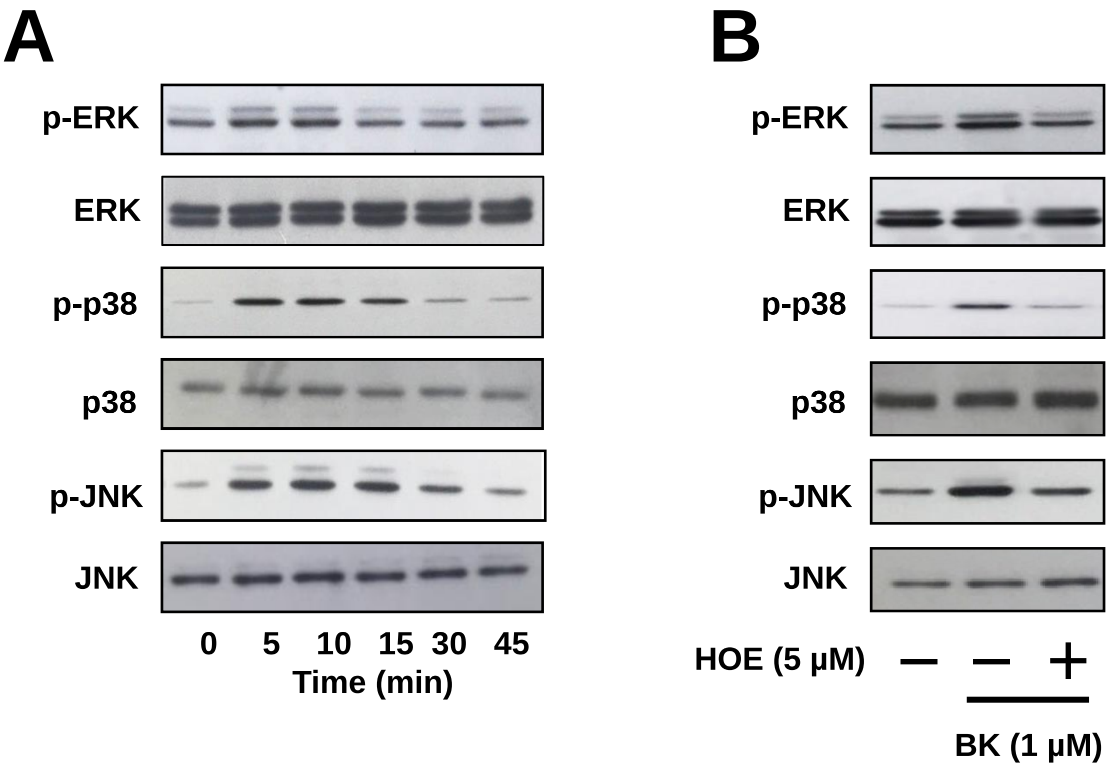

Supplementary Figure S3. Time-dependent bradykinin-mediated MAPK activations in U87-MG cells were sensitive to HOS-140 antagonism on B_2R**.** U87-MG cells were stimulated with increasing duration (0 – 45 min) of bradykinin (1 μM), all proteins in the subsequent cell lysates were resolved in SDS-PAGE, followed by immunodetection with various MAPK subgroups. (A) Bradykinin-induced rapid stimulatory phosphorylation of ERK, p38, and JNK, peaked around 5 – 10 min post-treatment and then gradually decreased afterwards. (B) Bradykinin-induced ERK, p38, and JNK activations were likely to be suppressed by B_2R-specific antagonist, HOE-140.

Supplementary Fig. S4

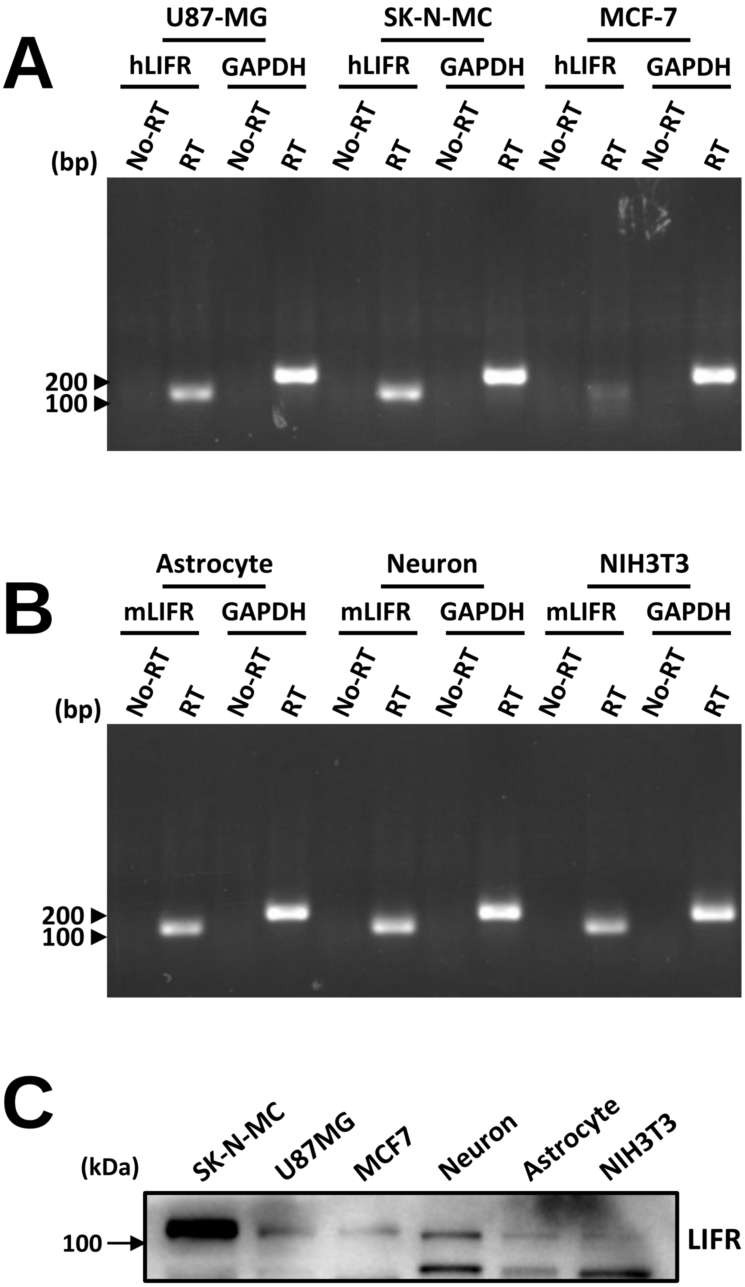

Supplementary Figure S4. The mRNA transcripts and expressed proteins of LIFR can be readily detected in human astrocytic cells and neuronal cells, as well as in murine primary astrocytes and neurons. Detections of mRNA transcripts of (A) human LIFR (hLIFR) in astrocytic U87-MG cells and neuronal SK-N-MC cells, and (B) murine LIFR (mLIFR) in primary astrocytes and primary neurons were performed by reverse transcription PCR associated with specific primers. GAPDH signals served as sample loading controls, and those samples subjected to PCR without initial reverse transcription (No-RT) served as the negative controls. Human MCF-7 cells (breast adenocarcinoma) and murine NIH-3T3 cells (embryonic fibroblast) were used as additional references for hLIFR and mLIFR detections, respectively. (C) Cell lysates (with 25 µg of protein per samples) of human origin (U87-MG cells, SK-N-MC cells, MCF-7 cells) and murine origin (NIH-3T3 cells, primary astrocytes and primary neurons) were subjected to SDS-PAGE, followed by immunodetection of endogenously expressed LIFR with a specific antibody.
